# Successful Moderation in Online Patient Communities: Inductive Case Study

**DOI:** 10.2196/15983

**Published:** 2020-03-17

**Authors:** Tanner Skousen, Hani Safadi, Colleen Young, Elena Karahanna, Sami Safadi, Fouad Chebib

**Affiliations:** 1 Management Information Systems Terry College of Business University of Georgia Athens, GA United States; 2 Mayo Clinic Rochester, MN United States; 3 Critical Care Medicine Fellowship University of Maryland Medical Center Baltimore, MD United States

**Keywords:** online patient communities, online social support, online community moderation, community management

## Abstract

**Background:**

Online patient communities are becoming more prevalent as a resource to help patients take control of their health. However, online patient communities experience challenges that require active moderation.

**Objective:**

This study aimed to identify the challenges of sustaining a thriving online patient community and the moderation practices employed to address the challenges and manage the online patient community successfully.

**Methods:**

An inductive case study of Mayo Clinic Connect was analyzed using the grounded theory methodology. Insights for the analysis were obtained from semistructured interviews with community managers and community members. Secondary data sources, such as community management documents, observational meeting notes, and community postings, were used to validate and triangulate the findings.

**Results:**

We identified four challenges unique to online patient communities. These challenges include passion, nonmedical advice, personal information, and community participation. We identified five categories of practices that community members used to address these challenges and moderate the community successfully. These practices include instructive, semantic, connective, administrative, and policing practices.

**Conclusions:**

Successful moderation in online patient communities requires a multitude of practices to manage the challenges that arise in these communities. Some practices are implemented as preventive measures while other practices are more interventive. Additionally, practices can come from both authority figures and exemplary members.

## Introduction

### Background

Patients increasingly rely on the internet to look for medical information; ask questions; find peers with similar health concerns; read commentaries and experiences about health issues; and consult reviews and rankings of treatments, doctors, and hospitals [1-4]. According to a 2013 study, 72% of internet users looked at health information online [5]. Online patient communities are becoming forums for patients to share their stories, gain peer support, and search for medical information [6,7]. Studies have shown that membership in patient communities is associated with better health, behavior, and medical knowledge [7-10].

We define successful online patient communities as communities that are vibrant, supportive, and active. They welcome members, encourage active participation and interactions, foster relationships, and provide accurate medical information. Among the many potential concerns and challenges for online patient communities, the quality and accuracy of medical knowledge provided in these communities are questioned [11-14]. This is especially important given that members vary considerably in their medical expertise and may have difficulty discerning information quality. To address these issues and assure their success, some online patient communities use moderators [15]. Little academic research exists on effective moderation practices in online patient communities. Our objective was to systematically study the challenges of online patient communities and identify moderation practices that are employed to address these challenges and maintain a successful community.

To gain a deep understanding of this phenomenon, we conducted an inductive case study of Mayo Clinic Connect, a leading online patient community. Using the grounded theory methodology, we collected observational and interview data from key members over 3 years. Our findings highlighted five moderation practices in the community: instructive, connective, semantic, administrative, and policing. Furthermore, these moderation practices were not only enacted by the community’s management but also by the enlisted volunteer community members who assist in achieving community success. Together, the practices used by community management and community members successfully address several challenges to create a thriving community.

### Prior Work

Online communities bring together members who share common interests and contribute valuable knowledge and expertise and are recognized as generators of significant knowledge [16]. In health care, online patient communities have become a medium for patients to share their stories, gain peer support, and search for medical information [6]. A healthy community retains its existing members, attracts new ones, and elicits their contribution [17]. Research has consistently shown the importance of feedback in driving members’ contributions to online communities [18-20]. One challenge facing online communities is managing the sheer number of contributors and contributions in the absence of monetary incentives and formal managerial structures [21].

Compared with other online communities, online patient communities face additional unique challenges. First and foremost, the quality, accuracy, and trustworthiness of medical knowledge provided over these platforms are questioned [11-14]. Second, there are ethical and privacy issues related to sharing and disseminating patients’ information online [22]. Third, there is a mismatch of motives and expectations among members who use these platforms as communication and marketing tools and members who use them for social support and exchanging advice [23].

Community leadership plays an essential role in dealing with online community challenges [20,24,25]. Effective leaders and moderators are characterized by inclusivity, helpfulness, and sociability [20,26,27]. Community management can be assisted by elevating the status of exemplary members to become trusted volunteer peer leaders [19]. In online patient communities, the involvement of volunteer peer leaders and echoing the voice of patients are essential for community success [28]. However, this inclusivity should not ignore the quality of information and the trustworthiness of conversation. Such tension is not very prevalent in other grassroots communities because the conversation reflects the shared interests of members without much further consideration. However, understanding how online communities can be other accurate sources of information and remain inclusive to members’ opinions remains an important research area [16,21].

## Methods

### Research Context

We conducted a study of Mayo Clinic Connect (referred to as Connect hereafter), a leading online patient community sponsored by Mayo Clinic. Connect is designed to “connect patients and family caregivers with each other” [29]. This community enables patients and their families with different medical backgrounds to share experiences, find support, and exchange information with others who have faced similar experiences. The community has open boundaries, which means it is open to the public and not restricted to Mayo Clinic patients only. At the time of publication, the community has 93,000 registered members and more than 10,000 active members who post at least once a month.

### Data Collection

Our primary source of data came from semistructured interviews that were collected over five phases. Levitt et al [30] claim that fidelity to data “may be procured by inviting participants to interview or describe their experiences.” First, data collection was initiated when 1 author became acquainted with the director of Connect and participated in an early interview with her. This interview was instrumental in establishing our initial understanding of the community and in guiding our sampling of subsequent interviewees through a snowballing approach [31]. Second, we conducted four interviews with managers of the community over the phone. Third, we attended a community meeting of managers and members who discussed the concerns, successes, and goals of the community. In this meeting, we participated as silent observers to understand the community in general and collected four onsite interviews from community members who were participating.

After the initial analysis of these interviews, we focused on theoretical sampling for subsequent interviews. Therefore, our fourth phase included the collection of additional 13 phone interviews from members of the community. Through probing questions, we asked about their participation in Connect and what they felt the community did to achieve success. We asked them to expound on their experiences with the community, including challenging ones. Overall, we let all interviewees talk about being a member of Connect and their interactions with others. Each interview lasted approximately 30 min and was recorded and transcribed verbatim. We continued to interview individuals until we reached theoretical saturation and gained no additional insights from additional interviews [32]. Fifth, we conducted a final interview with the director of Connect to discuss and validate our findings.

After the completion of the interviews, we supplemented this primary data source with additional observational data sources to triangulate the findings from the interviews. First, all authors joined the community as passive members and observed the community members’ contributions and interactions over the last 3 years. Second, we obtained archival community documents, which included standardized response templates, a reference guide for exemplary posts, orientation packages, and community management guidelines. The observations gathered from these data sources were in line with the findings that emerged in the interviews, strengthening the fidelity of the findings.

### Data Analysis

We adopted a grounded theory coding perspective on the semistructured interviews to let theory emerge from the data. Our initial unfamiliarity with the community was instrumental in allowing the data to inform our insights, rather than preconceived theory. However, we were aware of how our prior experiences as researchers might shape the interpretation of the data [33]. Therefore, as an added measure to ensure the coding was grounded in the data, the author who coded the majority of the data was the least experienced in online community literature. This perspective management in data analysis enhanced the fidelity of our data [30]. We followed a constant comparative process throughout our analysis, that is, we repeatedly compared codes and findings in frequent discussions among the authors [34].

Furthermore, 2 of the authors conducted line-by-line open coding techniques [35] in the first five interviews. After both the authors completed the coding of these first interviews, they discussed their findings to agree on emergent themes. One author coded the remaining interviews, and all authors met frequently during the coding process to discuss the inductive insights from the codes. After creating an extensive list of open codes, we conducted a second round of coding to ensure that minor differences in codes from open coding were combined to form unique ideas that did not overlap in the data. During open coding, memos were kept on each interview to provide extended notes and insights.

After completing open coding and discussing emergent themes, we followed an axial coding process to combine and relate codes together to form more abstract themes and relationships [36]. Axial coding helps to identify the who, what, when, where, why, and how of the emerging theory. In our case, it illustrated a clear picture of the moderation practices in the community.

We felt that the results from interview data were sufficient to generate insights, yet we used the observational data of archival documents and community postings to validate those results. Although this observational data were not formally coded in the same manner as the interviews, it was used as a source to validate the findings and provide examples of our presented results.

## Results

### Community Challenges

Our interviews highlighted certain challenges for online patient communities, specifically that require active moderation to address. These challenges include passion, giving nonmedical advice, providing personal information, and community participation.

First, many members are passionate about Connect and their contributions to it. These members experienced or overcame medical conditions and are passionate to share their experiences and help others. Thus, passion can yield positive consequences, such as increased engagement and participation. Passion, however, leads many individuals to argue about different beliefs and opinions. Ugly and argumentative posts are at times a challenge for Connect:

We might have a member that comes in really opinionated.

Moderator 3

Second, sharing personal experiences is perhaps the overarching activity of members in Connect. Members with similar problems share experiences to encourage, inform, and support one another. Overall, this activity creates positive outcomes, and many members are very grateful for the feeling that they are “not alone.” However, challenges arise when experiences are viewed as or claimed to be “medical advice.” Members in the community are not medical practitioners but solely patients who have experienced a similar disease state. Therefore, nonmedical advice creates the potential for disappointment if the advice does not yield positive outcomes, or worse, legal repercussions if that advice causes further physical or emotional harm:

When it comes to alternative medicine, complementary medicine, that's where we run into problems. Because members are free to express their views, but when they advise their discussion group and they instruct them to stop taking traditional medications and go for homeopathy or herbal things, that's when we have to step in.

Moderator 4

Third, soliciting personal information is helpful for community members to know how to respond to other members. More information regarding a member’s condition and situation helps others relate to the member’s issues and provide appropriate support. Members often ask questions such as “how was this diagnosed,” “how long have you had the problem,” and “what kinds of treatment have you had.” However, sometimes this is taken too far. Violations to privacy may occur when a member shares excessive personal health information:

In a HIPAA sense, this individual is asking this person very specific questions on the day, the time and the results of a medical test, and I’m not sure that that's an appropriate place for a public discussion board.

Mentor 6

Fourth, community participation is necessary by definition for the community to thrive. Therefore, a lack of participation is a challenge to the community. Yet, perhaps, the more nuanced challenge is that the motivation or form of the participation can vary. For example, many members will come to the community only for answers to their questions or to find a way to schedule an appointment with Mayo Clinic, in contrast to joining to establish relationships and gain social support. When members do not participate in the community to establish relationships, the turnover of community members is high, which will hurt the community.

In addition to the four challenges mentioned above—passion, nonmedical advice, personal information, and community participation—we also noticed secondary challenges that either were not mentioned sufficiently in the interviews or were related to the main challenges addressed. These challenges included ambiguous identities, lack of physician interest, generational gaps, and technology challenges. However, these were secondary to the main story we saw in the data, and therefore, we will focus on the four main challenges for the duration of the paper.

### Moderation Structure in the Community

To respond successfully to these challenges, the community must maximize the potential positive benefits and minimize the potential negative outcomes of addressing them. In the case of Connect, these challenges are addressed by community management. The management team of Connect consists of a director and 5 staffers. These managers are referred to as moderators in the nomenclature of Connect. Moderators have set formal guidelines such as being careful when giving out medical advice, being respectful, and no commercial advertising. At the outset of the community, the moderators ran the community, moderated others’ posting, and provided the support to members who started to join the community.

As the community grew, moderators recognized the need to scale community moderation to maintain members’ contributions. At the same time, moderators noted that some members were taking increasingly active leadership roles. They identified these emerging peer leaders and invited some of them to help monitor the community. In the nomenclature of Connect, the emerging peer leaders selected by the moderators are called mentors. Once selected, mentors, who volunteer their time, are trained and guided by moderators to act in accordance with the guidelines of the community. Multiple training practices are implemented to onboard and train mentors (Table 1).

**Table 1:** Training mechanisms for mentors.

Training mechanism	Example from interviews
Standard documentation	“I'll send you three or four documents that you can look through and understand a little more what we're looking for in a mentor.”
Setting expectations	“We’re hopeful that you’ll agree to spend at least X number of hours a month visiting the board and watching things.”
Coaching and individual training	“When you are first invited, you spend time with the director, a one-to-one, doing screen shares and discussing different scenarios.”
Mentor meetups	“The mentors and moderators, we get together once a year. It is a little bit more training and sort of continuing education of being a mentor.”
Remote training	“Every quarter we will have a phone call where we can share what concerns us and build up a discussion.”
Behavior emulation	“We [Moderators] ask them to emulate us as far as the way we would respond.”

These training practices communicate and transfer the goals of moderators to mentors. This process brings three advantages. First, it reduces the workload of moderators, which is becoming infeasible with the continuous growth of the community, receiving around 10,000 posts per month. Second, mentors are uniquely qualified to assist members because of their shared experience—an experience that the moderators often do not have. Third, it enables moderators and mentors to specialize in different tasks focusing on desired outcomes. Moderators focus on administrative responsibilities, policing the community, and resolving tensions. Mentors can focus on patient success through the daily postings in community threads.

### Moderation Practices in the Community

Through the analysis of the interviews, we found that moderators and mentors enacted different practices to address the challenges of the community. Groups of similar practices were abstracted into five high-level categories to add parsimony to our understanding of the moderation. Although some practices are employed by *both* moderators and mentors, in general, practices were not shared equally between moderators and mentors. The three practice groups associated more often with mentors were instructive, semantic, and connective practices. The two practice groups associated more often with moderators were administrative and policing practices.

*Instructive practices* are actions that provide medical information relevant to member experiences. Although the community acts as emotional support for individuals, the community also strives to empower members by educating them on how to make better decisions about their health. Specific examples of instructive practices include pointing a member to a doctor, stating that the community is not a replacement for professional help. Mentors not only point people to doctors but also assist them by sharing appropriate questions that could be asked or tips of ways to talk to a doctor. In addition, mentors in the community have become proficient in using tools such as Google Scholar to search and provide relevant links to patients who have medical-related questions, inspiring them to learn for themselves through peer-reviewed literature. At times, moderators invite doctors to participate in Connect through live webinars that answer common questions that patients have asked recently.

*Semantic practices* are actions that focus on the content of messages and responses, that is, what is being communicated in a post. Mentors are instructed to share beneficial content in addition to sharing it in a caring and empathetic manner. Some of the beneficial content that mentors can post are their own experiences with past illnesses that relate to patients and show them that they are not alone in their journey. Mentors can also post words of welcome to new members to establish a sense of security, love, and care. In addition, mentors ask questions to spur further discussion and better understand the individuals they are communicating with. The content of a message, however, is only half of the meaning of semantic practices—how the message is delivered is also important. Mentors must, when appropriate, validate the concerns of the posts through their words to make patients feel heard and important. This is enhanced when mentors use encouraging language and positive reinforcement, rather than critical and negative comments that are so often seen online.

*Connective practices* are actions that create relationships inside the community and bring individual members into a community setting. This connection is achieved through the platform design of *tagging* members as seen in many social media sites. Mentors go beyond tagging, however, and take a proactive approach to create relationships. Two examples include proactively following up with individuals who have not posted recently and attempting to connect them with similar members. In addition, mentors who receive private messages (a feature on Connect) will often ask to bring them public so that the entire community can see the questions and participate in supporting that individual. So, although the feature of tagging is often how connections are made, the proactive mindset to foster relationships is a significant element for connective practices.

*Administrative practices* are performed by moderators outside of discussion threads to keep the community growing with a *positive community feel*. What is done behind the scenes plays an important role in creating an environment for the community discussion to stay vibrant and productive. Administrative practices include increasing the number of volunteer mentors to help scale the moderation of the community. Once mentors are selected, moderators coach them on the proper conduct in the community. In addition, moderators will, at times, coach regular members who act inappropriately. Finally, moderators of the community attempt to build community unity by spotlighting select members frequently.

*Policing practices* are interventions against antisocial behaviors and departures from community guidelines. Ideally, the community would like to not have to implement these practices, but because of the sensitivity of medical information, they are implemented when needed. Moderators act as the authoritative hand more than mentors. Mentors may indirectly police the actions of members by asking for evidence of claims or diffusing negative and criticizing remarks, but typically, they are used as eyes on the community to report posts. Moderators alone implement policing interventions through muting members, taking down posts, and acting as an intervention when posts get out of hand.

### Addressing Challenges Through Moderation Practices

After we independently identified specific challenges and moderation practices of Connect, we used axial coding to relate the challenges and practices together. By doing so, we uncovered processes by which mentors and moderators worked together to maximize the benefits that come from challenges and minimize their negative impacts. Below, we identify each challenge that was introduced above and discuss the practices implemented by moderators and mentors to address the challenge. We highlight the insights with relevant excerpts that came from interviews of community members.

#### Passion

Passion was fostered by mentors showing interest in members’ posts. Particularly, semantic practices, such as asking questions and validating concerns, were used to leverage the positive benefit of passion and expound on the member’s interest. When a member’s passion turned negative and argumentative, however, mentors addressed this challenge through indirect policing practices such as diffusing negative comments and observing the community and reporting to moderators:

We’ve had our share of those people who come in and are negative, and as a mentor, I honor that because we’re not all going to be positive every day. I will recognize that and say, “Hey, it sounds like you’re having a really bad day. Is there anything that caused that?”

Mentor 8

We had a member who was really opinionated, and couldn’t be convinced he was giving bad advice, so in the end, what I had to do was call on the director to take a hand in the matter.

Mentor 4

However, when the discussion became too ugly, moderators would intervene and address the situation. Moderators typically did not remove posts or mute members who shared passionate opinions because they recognize that passion is what strengthens the community. Rather, moderators implemented administrative practices such as coaching to work with members and correct members’ contribution to the community:

And there was one particular gentleman who was very combative, talked down to members a lot, wrote in all caps, which in an online community, is considered yelling. And with a lot of coaching, that particular person, over the course of many months, ended up becoming a really valuable member.

Moderator 2

If members did not respond to coaching or if the negative behavior continued, then direct policing practices such as muting members or removing posts were implemented as a final option:

In one case, the moderators actually did ban the person from the board and say, “We don’t allow people in here who are going to call people names and take things to that extreme.”

Mentor 6

#### Nonmedical Advice

Giving *advice in a proper way* is a manner in which the community achieves its goals of educating and supporting patients. In lieu of advice, mentors share their experiences, a semantic practice, which follows the guidelines of Connect. Their post with their experience not only benefits the patient in need but also, hopefully, acts as a model for others to follow. They attempt to include the phrase, “this is not medical advice” before each of their experiences:

You definitely want people to realize that I’m not a doctor. But you want to be able to offer help to people. So, we have to work in certain ways. And I usually just say, “based on my experience, this is what happened.”

Mentor 14

In addition, mentors have been instructed on how to question nonmedical advice by asking, “Where did you find that information?” They use instructive practices that educate members on how to do their research to find valid medical information and sometimes share those sources with patients:

When I see something that I am not familiar with I will definitely say, “I am not familiar with that. Have you checked that research? And we do have guidelines to help refer people to approved sites.”

Mentor 5

Moderators invite doctors to participate in live webinars to provide a professional answer regarding questions that have spurred nonmedical advice. Hopefully, these webinars provide the needed answers that the community can refer to when they encounter an instance of nonmedical advice. However, moderators will use policing practices as well to intervene in discussion threads if there is any information shared that could be harmful to patients:

We will do a live Q&A session between a physician and the surgeon. Members are able to ask questions prior. They can watch the video live, and then we link that to Mayo Connect so that the information is housed on Connect too.

Moderator 3

There are times where it is bad advice. That’s where we step in, in those situations. And we’ll, I wouldn’t say squash the conversation, but relay how important it is to seek an appointment with a medical provider.

Moderator 2

#### Personal Information

To gather information to help patients, mentors will use semantic practices to validate concerns and ask questions to members. These questions prompt further discussion and deeper insights that allow the community to help a member’s concerns. Many members, however, do not understand the proper information to divulge and to retain when they join the community. Mentors will help new members with welcome messages that establish expectations, often teaching them what is appropriate, what are the goals of Connect, and who are some of the other people in the community:

I try to relate empathically to what they’re going through. That makes it easier to discuss how we feel, or have felt in a similar situation.

Mentor 12

Asking questions to the person is a good thing. I know that people don’t always like to post on a public forum, so I always start a sentence with, “if you are comfortable sharing more information about yourself, would you please tell us...etc.”

Mentor 7

If they’re brand new, I welcome them to the site and explain a little bit how it works.

Mentor 9

Moderators will promote the sharing of personal information by spotlighting members. These spotlights are a good example of personal information that is appropriate to share to help others know how to communicate with a patient. Moderators also use the administrative practice of coaching to help instruct members when they are sharing too much. In addition, moderators use policing practices of deleting posts to remove information that is potentially Health Insurance Portability and Accountability Act of 1996 sensitive:

We do a spotlight to get to know people personally. Their favorite things, where they live, what they like to do, and beyond having a condition, that we are people too.

Mentor 8

You might not want to give all that information and I’ve had to refer them to the director and say, “There is a little too much information shared here. Let’s talk to this person, edit their post, and take out the exact department building where they are living.”

Mentor 8

#### Community Participation

Mentors enact multiple practices to maintain member participation. Participation includes the amount of content that a user posts, but it can also signify if a member remains involved in the community. Mentors help to increase the amount of participation by semantic practices, namely, asking questions and welcoming members. Mentors additionally focus on retaining members through connective practices, particularly tagging members to foster relationships and proactively following up with patients who have not been heard from recently:

I think you just ask a lot of open-ended questions. Usually, I say, “I’m fascinated by what you’ve told us, please tell us something else.”

Mentor 10

If a member has been posting and then suddenly stops, we tag members to connect them to our members.

Moderator 4

Moderators also implemented connective practices in the same manner as mentors, without a major distinction between roles. Our interpretation is that moderators of the community were the initial *mentors* of the community when the community was in its early stages, and those moderators have continued to maintain the relationships that were created then. In addition, moderators implement connective practices to model how to properly tag members:

When I first started, it was really more about fostering relationships and, no pun intended with the word connect, but in the beginning really our job was to connect members with other members who had talked about the same thing.

Moderator 2

**Figure 1:**
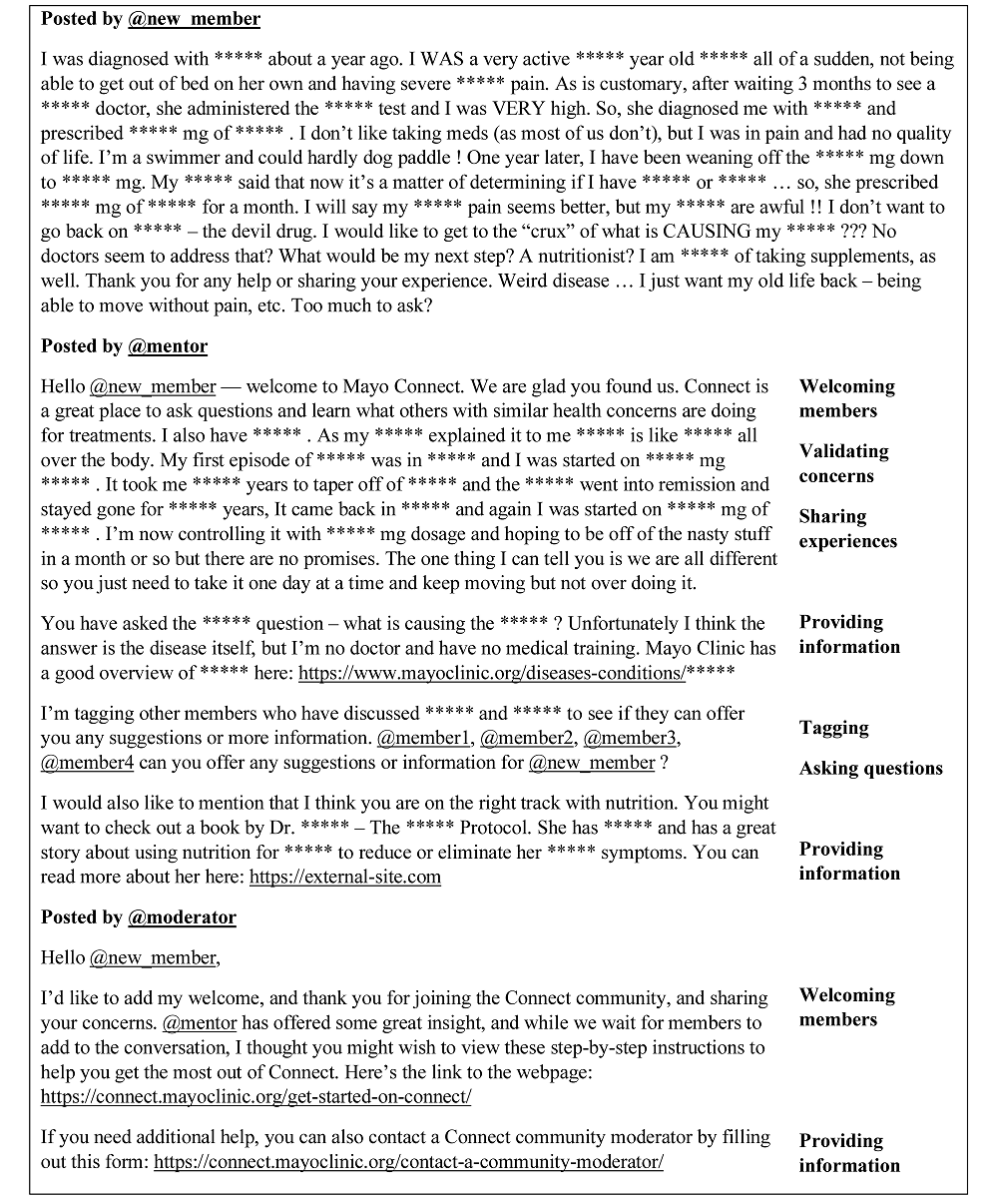
Moderation practices employed in one example post.

**Figure 2:**
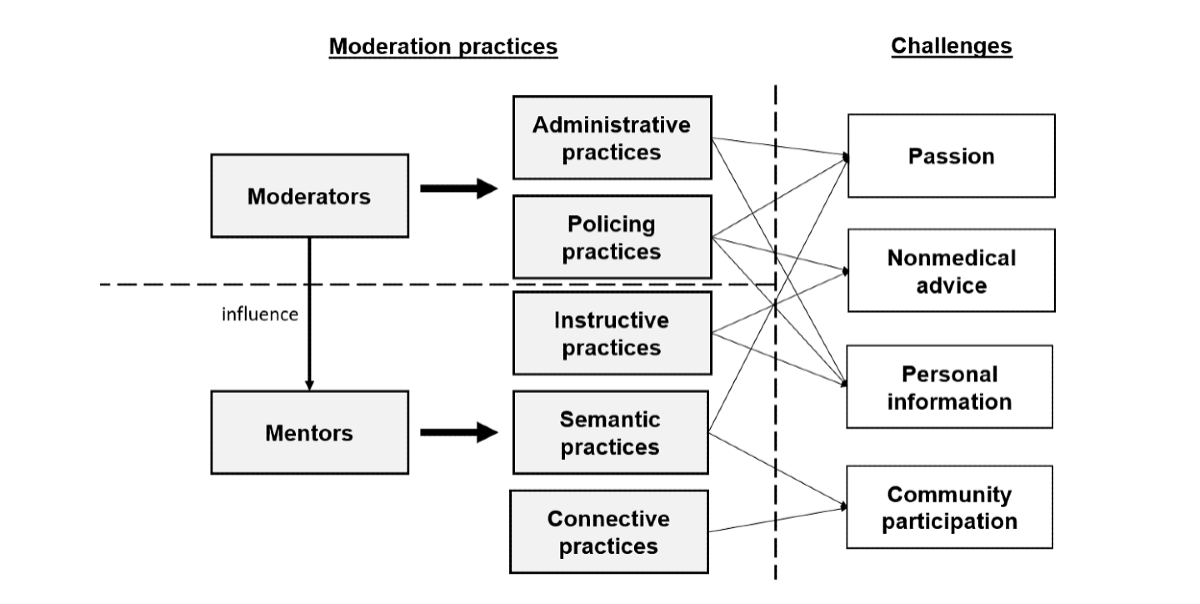
Graphical summary of our findings.

We provide 2 figures to help readers understand the context and the findings. First, in Figure 1, we include a redacted screenshot of a post from Connect to illustrate the practices implemented by moderators and mentors. We highlight some specific semantic, connective, and instructive practices to the right. However, our main purpose for including the screenshot was for readers to form their own opinions of the community and familiarize themselves with the context. Second, in Figure 2, we provide a graphical summary of the roles that implement the practices, the five main practices identified, and the four challenges that those practices address. We provide this graphic to help quickly and visually outline the dynamics of the community.

## Discussion

Like most online communities, Connect experiences multiple challenges. We have identified and explicated many practices jointly used by Connect moderators and mentors to address these challenges and maintain a thriving community. We have provided a graphical summary of our findings in Figure 2. These practices are not solely emergent or wholly prescribed. The nature of the practice matches the needs of the community and its members. Some of these practices are *interventive*. They are mostly used to provide a quick and direct resolution to time-sensitive challenges. Other practices are *preventive*. They promote members’ behavior that leads to desired outcomes by avoiding tensions. These practices can be a guide and reference for an online patient community to promote the success and well-being of its members.

Beyond the context of health care, these findings illustrate how knowledge-creating online communities [16] can balance the need to promote the contribution of accurate and trustworthy knowledge with the need to remain inclusive to members’ opinions and interests. Such an endeavor requires the investment of time and effort to promote a culture of trust and the joint stewardship of the community by its management and volunteer members. Finally, this research presented the findings of one case study. Future work can improve the generalizability of the findings to other online communities in other contexts.

## Abbreviations

MIS: management information system
